# Clearance of corticosteroids in pediatric patients with active eosinophilic esophagitis: Faster than expected

**DOI:** 10.1111/pai.70377

**Published:** 2026-06-10

**Authors:** Pamela Fernanda Alves Barbosa, Marcelo Tatit Sapienza, Antonio Carlos Pastorino, Ariana Campos Yang, Glauce Hiromi Yonamine, Mayra de Barros Dorna, Ana Paula Beltran Moschione Castro

**Affiliations:** ^1^ Clinical Immunology and Allergy Unit, Department of Pediatrics, Children's Institute, School of Medicine University of Sao Paulo Sao Paulo Brazil; ^2^ Nuclear Medicine Unit, Department of Radiology, Radiology Institute, School of Medicine University of Sao Paulo Sao Paulo Brazil; ^3^ Division of Allergy and Immunology General Hospital of the Medicine School of University of Sao Paulo Sao Paulo Brazil

**Keywords:** budesonide, corticosteroid therapy, eosinophilic esophagitis, esophageal transit scintigraphy, oral viscous budesonide, swallowed topical corticosteroid, topical corticosteroid therapy

To the Editor,

Eosinophilic esophagitis (EoE) is a chronic, immune‐mediated inflammatory disease, characterized by symptoms of esophageal dysfunction and histological evidence of ≥15 eosinophils per high‐power field on esophageal biopsy, excluding other causes of esophageal eosinophilia. In younger children, manifestations include abdominal pain, vomiting, failure to thrive, food refusal, feeding difficulties, and adaptive eating behaviors. In older children and adolescents, dysphagia can range from mild to severe, making EoE a leading cause of food impaction in pediatric patients.^1^

Swallowed topical corticosteroids (STC) are a first‐line therapy for EoE.^1^ These treatments often involve medications originally developed for asthma or allergic rhinitis, either administered directly to the oropharynx and then swallowed, or thickened with vehicles to increase esophageal mucosal contact, such as in oral viscous budesonide (OVB).^2^ OVB has demonstrated efficacy in clinical trials and systematic reviews and is strongly recommended in guidelines.^1^, ^3^

New STC were developed for EoE treatment aiming to further optimize mucosal contact time, showing greater efficacy and high histologic remission rates.^4^, ^5^ However, these are not yet approved across all pediatric age groups and remain unavailable in many regions.^3^ Thus, OVB continues to be necessary. Nevertheless, their esophageal contact time in pediatric patients with active EoE is unexplored, especially considering that inflammation from active EoE may affect esophageal motility, potentially altering clearance and retention of OVB.

This was a prospective, controlled, crossover study involving pediatric patients with active EoE and evaluated esophageal clearance of two OVB formulations—Preparation A and B—using esophageal transit scintigraphy (Figure 1). Preparation A used 2 mL of budesonide inhalation suspension (500 mcg/2 mL), thickened with 1.8 g of powdered sucralose (standard formulation). Preparation B mixed 400 mcg of inhaled budesonide powder with 3 mL of corn syrup. In both, 0.3 mL of radiotracer (0.5 mCi technetium–99 m phytate) was added, yielding a total of 4 mL for oral administration.

**FIGURE 1:**
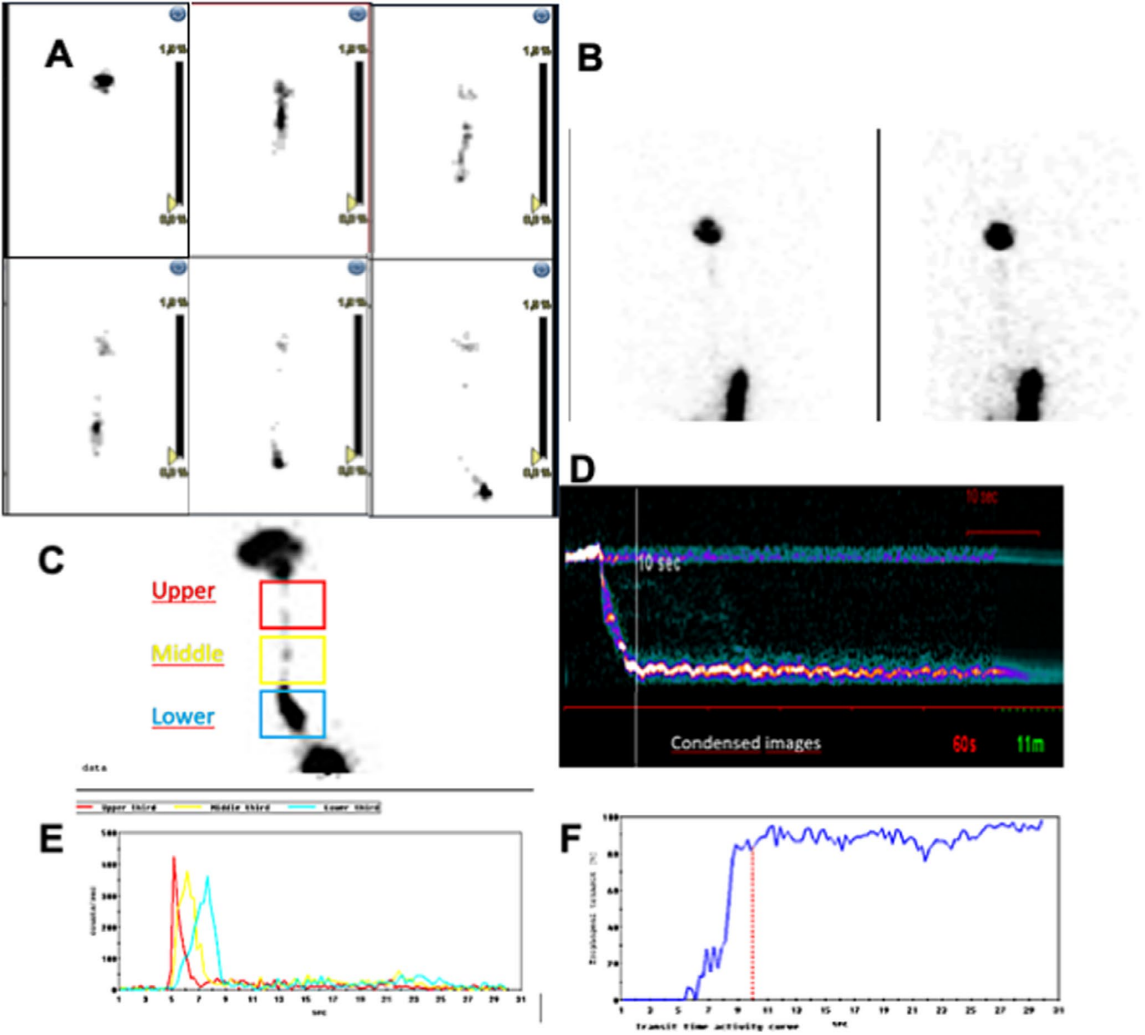
Esophageal transit scintigraphy performed in a study patient. (A) Posterior upright dynamic images. (B) Posterior upright composite image showing esophageal activity throughout the test. (C) Regions of interest (ROIs) defined for the upper, middle, and lower thirds of the esophagus. (D) Time‐activity curve generated from condensed images (each image corresponds to one column). Progressive transit from the oral cavity to the stomach occurred within the first 10 s. (E) Time–activity curves of the upper, middle, and lower esophageal segments. (F) Cumulative curve displaying total activity that passed through the esophagus over time.

The primary outcome was mucosal contact time, measured by the area under the curve (AUC) during the first 3 min post‐ingestion. Secondary outcomes included: (i) esophageal transit time (ETT), defined as the time between the arrival of 50% of peak radiotracer activity in the proximal esophagus and its retention at 50% across the esophagus; (ii) esophageal emptying time (EET), defined as the interval between the same starting point and the clearance of 90%; (iii) percentage clearance at 10 s after peak activity; and percentage residual activity at (iv) 10, (v) 20, and (vi) 30 min post‐administration. Taste and texture were rated using a validated visual analog scale after each exam.

Patients were recruited from a single EoE referral center between February 2020 and March 2024 and the recruitment was paused during COVID‐19 pandemics. Each patient underwent two scintigraphy sessions, 7–14 days apart, ingesting one preparation per session. Both were prepared and administered by the same evaluator. Imaging followed a standardized protocol used for achalasia. All scans were interpreted by the same nuclear medicine physician (see Appendix S1 for full methodology).

Twelve patients completed the study (7 females, 5 males), including 9 adolescents. The median age was 12 (range: 7–17) and the median disease duration was 2.5 years (range: 0–8). Seven patients showed endoscopic signs of fibrostenosis (linear furrows, trachealization, or strictures), and the median peak eosinophil count on esophageal biopsy was 44 eos/hpf (range: 16–106). Ten were receiving OVB at the time and only two patients had no history of IgE‐mediated food allergy.

Esophageal clearance parameters for both formulations are summarized in Table 1 and Table S1. All variables were comparable between formulations A and B, with no statistical differences. Palatability scores were also similar, with median values of 7 and 8, respectively (*p* = .47). Figure 2 compares AUC between formulations A and B for each patient.

**TABLE 1:** Comparison of esophageal clearance parameters between formulation A and B.

Esophageal clearance parameters (*n* = 12)^a^	Formulation A	Formulation B	*p*
Total AUC Median (min–max)	14,191 (8119–42,139)	13,398 (4907–35,150)	.26
Total AUC Median (min–max)	2896 (1769–17,316)	3505 (1234–13,202)	.38
Medium AUC Median (min–max)	3505 (2101–14,169)	3664 (1245–9528)	.38
Inferior AUC Median (min–max)	7105 (3026–17,866)	6344 (1471–20,868)	.38
ETT (s) Median (min–max)	4.8 (2.2–8.8)	4.9 (3.1–12.3)	.8
EET (s) Median (min–max)	7 (4–17)	12 (4–26)	.22
% Clear 10 s max (%) Median (min–max)	82.5 (46.6–95)	79 (49–97)	.37
% Ret 10′ (%) Median (min–max)	0.7 (0.3–2.7)	0.7 (0.3–1.3)	.41
% Ret 20′ (%) Median (min–max)	0.4 (0.2–1.9)	0.4 (0.2–0.6)	.23
% Ret 30′ (%) Median (min–max)	0.4 (0–1.7)	0.5 (0.2–0.5)	.8

*Note*: % Clear 10 s max: Clearance percentage 10 s after peak activity; % Ret 10′, 20′ and 30′: Residual Activity at 10, 20, and 30 min.

Abbreviations: AUC, area under the curve; EET, esophageal emptying time; TET, total esophageal transit time.

^a^ For the calculation of segmental AUCs (superior, medium, and inferior thirds), data from 11 patients were analyzed, as one participant swallowed the preparation before the imaging command and was excluded from this part of the analysis.

**FIGURE 2:**
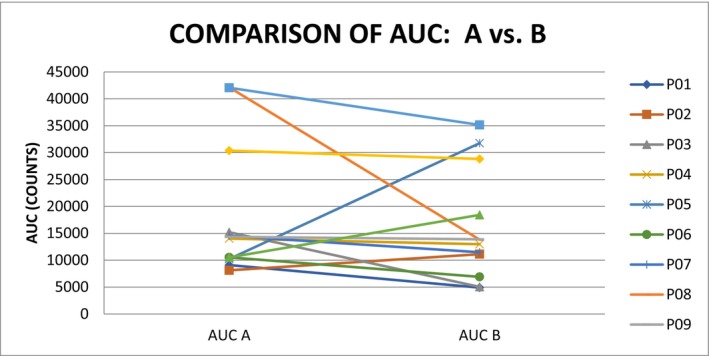
Comparison of area under the curve between formulations A and B for each patient.

STC has become a cornerstone and stands as the most extensively studied treatment option in the management of EoE. Nevertheless, there is considerable heterogeneity in the way OVB are prepared, with variations in ingredients, concentration, volume, and thickness.^3^, ^4^ Through this study, we were able to document the esophageal clearance of two OVB formulations in pediatric patients with active disease, an investigation not previously reported.

Analysis of OVB esophageal clearance has been described in prior studies involving adults with and without EoE,^2^, ^6^ but these evaluations may not necessarily be extrapolated to pediatric patients. Comparisons across these studies may be limited by methodological differences, including radiotracer type and dose, radiation exposure, the volume of swallowed formulation, and intrinsic characteristics of pediatric patients that may differ from adults such as medication acceptance, esophageal length, and motility. These factors highlight the importance of interpreting our findings cautiously.

In this context, certain variables provided a more tangible assessment of esophageal emptying velocity, which was notably rapid. ETT was particularly informative with median values of 4.8 s for Preparation A and 4.9 s for Preparation B, which is consistent with previous findings in healthy children aged 4–16 undergoing liquid ingestion (ETT = 4.6 s).^7^ High emptying percentages were also observed within 10 s after peak radiotracer activity, and less than 1% of the radiotracer remained in the esophagus 10 min post‐ingestion, further confirming rapid medication clearance.

The observed comparability between vehicles carries practical implications for local referral centers. In Brazil, powdered budesonide is significantly more affordable than its liquid counterpart. Demonstrating equivalence—particularly to a formulation recommended in the literature—may improve access, especially while specifically developed topical corticosteroids are not yet approved.

Another important consideration regarding the rapid esophageal clearance observed is whether the effect of these formulations is truly predominantly topical or systemic. The consistent demonstration of clinical efficacy, coupled with the low frequency of systemic adverse effects despite the administration of relatively high doses of swallowed corticosteroids, supports a primarily topical mechanism of action.^5^, ^8^

Finally, acceptability is a key factor in long‐term treatment adherence in pediatric populations. Both formulations were well tolerated in terms of taste and texture.

The esophageal transit scintigraphy technique proved to be a useful and feasible tool for evaluating esophageal clearance in pediatric patients with EoE. It offers several advantages, including low radiation exposure (approximately 0.3 mSv), a reproducible protocol, and no need for sedation. The procedure was well tolerated by children as young as 7 years old. This technique is already used in pediatric populations to assess motility disorders such as achalasia and scleroderma, and it enables the evaluation of various bolus consistencies, from liquids to solids, with patients in different anatomical positions, thereby allowing a more physiologic simulation than other tests.^9^

There are, however, some limitations inherent to the scintigraphic procedure itself. Operational issues during image acquisition may affect the accuracy of the results. For example, one adolescent patient swallowed the preparation before the command was given to start image capture, which compromised the ability to analyze the AUC for the esophageal segments. Other limitations of this study include its single‐center design and small sample size, although it was comparable to those in previous studies, mainly due to the difficulty in scheduling two closely spaced visits for the scintigraphic exams.

EoE is increasingly recognized as a relevant condition in pediatric practice. It is a chronic disease that requires long‐term management. Although new therapies have emerged, OVB remains effective, accessible, safe, and widely adopted in clinical settings. Understanding the esophageal clearance profile of these formulations contributes to more precise and evidence‐based therapeutic use. However, ensuring equitable access to effective SCT at an affordable cost remains a key priority to reduce treatment disparities worldwide.

## AUTHOR CONTRIBUTIONS

**Pamela Fernanda Alves Barbosa:** Writing – original draft; conceptualization; investigation; methodology; formal analysis. **Ariana Campos Yang:** Resources; writing – review and editing. **Ana Paula Beltran Moschione Castro:** Conceptualization; writing – review and editing; methodology; formal analysis; supervision; writing – original draft. **Marcelo Tatit Sapienza:** Methodology; formal analysis; software. **Antonio Carlos Pastorino:** Writing – review and editing; resources; supervision. **Glauce Hiromi Yonamine:** Methodology; writing – review and editing. **Mayra de Barros Dorna:** Writing – review and editing.

## FUNDING INFORMATION

The study received no external funding.

## CONFLICT OF INTEREST STATEMENT

The authors declare no conflicts of interest related to this work.

## DISCLOSURE

This study was conducted as part of the Master's degree research project of the corresponding author at the Department of Pediatrics of University of Sao Paulo, Sao Paulo, Brazil.

## ETHICS STATEMENT

All procedures were approved by the institutional ethics committee (approval number: 3.830.042) and conducted in accordance with the Declaration of Helsinki.

## CONSENT

Written informed consent was obtained from the parents or legal guardians of all participants, and assent was obtained from children aged 7 and older.

## Supporting information


Appendix S1.

Table S1.

## Data Availability

Data sharing not applicable to this article as no datasets were generated or analysed during the current study.
