# Supplementary material for: Clearance of corticosteroids in pediatric patients with active eosinophilic esophagitis: Faster than expected
Source: Pediatr Allergy Immunol. 2026 Jun 10;37(6):e70377. doi: 10.1111/pai.70377 (PMC13253919; doi:10.1111/pai.70377)
Supplement: Supplementary file 1 — Appendix S1. [file PAI-37-e70377-s001.docx]

**Supplement**

**METHODS**

The study was approved by the local Research Ethics Committee (number 3.830.042). A written informed consent form was obtained from all patients and their parents for their participation in this research.

**Inclusion/exclusion criteria**

This study included pediatric patients aged 7–17, diagnosed with active Eosinophilic Esophagitis (EoE). Patient selection occurred between 2020 and 2024, with patients that attended the Allergy and Immunology Units of Clinical Hospital of the University of Sao Paulo, which is a national reference center for the treatment of EoE. The diagnosis of EoE was defined by clinical history with presence of at least one sign or symptom (abdominal pain, food impaction, dysphagia, vomiting, and/or poor weight gain or growth) and histologic features consistent with EoE, defined as >15 (eos/hpf) in esophageal biopsy specimens. Active disease was defined as the simultaneous presence of clinical symptoms and histologic findings compatible with EoE, both documented within four weeks prior to study enrollment via medical history and upper endoscopy with biopsy. Exclusion criteria included: use of systemic corticosteroids in the past 3 months; presence of gastrointestinal diseases other than EoE; history of allergy to the radiotracer or any component of the tested formulations and female patients with a positive pregnancy test at the time of study enrollment.

## **Esophageal Transit Scintigraphy Procedure**

Scintigraphy was conducted at the Nuclear Medicine Department of Radiology Institute of Hospital das Clínicas, University of São Paulo Medical School (InRad–HCFMUSP), in São Paulo, Brazil. Radiotracer dose was adapted to pediatric guidelines, minimizing exposure^1,2^.

At Visit 1, patients received the standard formulation (Preparation A), followed by esophageal transit scintigraphy. At Visit 2 (7–14 days later), they received an alternative formulation (Preparation B).

Patients fasted for 4 hours prior to the exam. Before administration, they practiced swallowing commands using 10 mL of water. The test was performed with patients seated, with the gamma camera field centered to include the oral cavity, esophagus, and stomach. After verbal instruction, patients swallowed the test bolus, and image acquisition began immediately.

Images were acquired using an NM Discovery 630 gamma camera (GE Healthcare, USA) with a 64x64 matrix. The dynamic phase lasted 1 minute at 4 frames/sec, followed by a 10-minute acquisition at 4 frames/min (15 sec/frame). A static 1-minute image was captured at 20 minutes post-administration; if radiotracer was still visible, another static image was taken at 30 minutes.

## **Image Processing and Analysis**

The same professional, a nuclear medicine radiologist at InRad–HCFMUSP, processed and analyzed all images to ensure consistency. A static composite image was created using the first-minute dynamic frames (frames 1–240). Regions of interest (ROIs) were delineated for the upper, middle, and lower thirds of the esophagus and applied across the dynamic sequence for analysis.

Radioactivity over time was measured across a 180-second interval, normalized to acquisition time and administered dose, and plotted as counts per second (y-axis) over time in seconds (x-axis), creating time-activity curves.

## **Esophageal Clearance Parameters**

Clearance parameters were derived from the time-activity curves, including:

**a)** **Area Under the Curve (AUC) – Contact Time:** AUC was calculated in counts, indicating relative mucosal contact time. Higher AUC reflects longer esophageal exposure. AUC was calculated for the entire esophagus and for each segment alone. Subgroup comparison included patients with and without fibrostenosis.

**b)** **Esophageal Transit Time (ETT):** The interval between the arrival of 50% of peak activity in the proximal esophagus and when 50% of that value remained across the entire esophagus, based on the method reported by Chojnowski and Bestetti^3,4^.

**c)** **Esophageal Emptying Time (EET):** The time between the arrival of 50% of peak activity and clearance of 90%.

**d) Clearance Percentage at 10 Seconds:** Proportion of radiotracer cleared 10 seconds after peak activity.

**e) Residual Activity at 10, 20, and 30 Minutes:** Percent radiotracer remaining at each timepoint. At 30 minutes, data were available for 11 patients.

## **Organoleptic Properties**

After each exam, the evaluator administered a standardized taste and texture survey using a validated child-adapted visual analog scale (1 = very bad to 5 = very good)^5^. Scores were recorded for both formulations for comparison.

Patients were followed throughout the entire exam by a trained research evaluator and returned to their standard clinical care afterwards.

## **Statistical Analysis**

Sample size estimation was based on a previous adult study showing a minimum detectable AUC difference of 20,000 counts (≈50% variation). A sample of 12 patients per formulation was sufficient for 80% power^6^. As this was a crossover design, each of the 12 pediatric patients served as their own control.

Descriptive statistics summarized demographic and clinical characteristics. Normality was tested using the Shapiro-Wilk test. Nonparametric variables were presented as medians with minimum and maximum; parametric variables as mean ± SD. The Wilcoxon signed-rank test compared paired nonparametric data, p-values <0.05 were considered statistically significant. Analyses were conducted in Jamovi (version 2.6, 2024).

**REFERENCES**

1. Fahey FH, Treves ST, Adelstein SJ. Minimizing and Communicating Radiation Risk in Pediatric Nuclear Medicine. *J Nucl Med*. 2011;52(8):1240-1251. doi:10.2967/jnumed.109.069609

2. Knight LC. Update on Gastrointestinal Radiopharmaceuticals and Dosimetry Estimates. *Semin Nucl Med*. 2012;42(2):138-144. doi:10.1053/j.semnuclmed.2011.11.001

3. Chojnowski M, Kobylecka M, Olesińska M. Esophageal transit scintigraphy in systemic sclerosis. *Rheumatology*. 2016;54(5):251-255. doi:10.5114/reum.2016.63666

4. Bestetti A, Carola F, Conciato L, Marasini B, TarÃ GL. Esophageal Scintigraphy with a Semisolid Meal to Evaluate Esophageal Dysmotility in Systemic Sclerosis and Raynaud’s Phenomenon.

5. Jorge IMDG. *Aceitação de alimentos por pré-escolares e atitudes e práticas de alimentação exercidas pelos pais*. Doutorado em Nutrição em Saúde Pública. Universidade de São Paulo; 2011. doi:10.11606/T.6.2011.tde-27042011-094341

6. Hefner JN, Howard RS, Massey R, et al. A Randomized Controlled Comparison of Esophageal Clearance Times of Oral Budesonide Preparations. *Dig Dis Sci*. 2016;61(6):1582-1590. doi:10.1007/s10620-015-3990-4
