# Supplementary material for: Clearance of corticosteroids in pediatric patients with active eosinophilic esophagitis: Faster than expected
Source: Pediatr Allergy Immunol. 2026 Jun 10;37(6):e70377. doi: 10.1111/pai.70377 (PMC13253919; doi:10.1111/pai.70377)
Supplement: Supplementary file 2 — Table S1. [file PAI-37-e70377-s002.pdf]

Table 1. Complete Data for all patients

Patients	AGE	TIME OF EOE (YEARS)	No EOS	TASTE A	TEXTURE A	TASTE B	TEXTURE B	TOTAL AUC A	TOTAL AUC B	SUP AUC A	SUP AUC B	Medium AUC A	Medium AUC B	Inf AUC A	Inf AUC B	ETT Prep A	ETT Prep B	EET A	EET B	A % 10s MÁX	B % 10s MÁX	RET 10' A	RET 10' B	RET 20' A	RET 20' B	RET 30' A	RET 30' B
P01	15	8	100	5	4	4	4	9100	4907	2311	1887	2101	1549	4687	1471	6,8	4,3	7	5	90	97	0,3	0,7	0,2	0,4	0,2	0,3
P02	12	3	80	3	2	4	4	8119	11122	2055	4377	3038	2494	3026	4251	4,1	3,6	6,5	26,6	88	85	0,5	1	0,4	0,4	0,3	0,7
P03	17	7	30	5	2	5	4	15206	5040	4056	1234	3645	1245	7505	2561	5	5,1	5,5	15,6	46,6	66	0,6	0,9	0,4	0,4	0,5	0,6
P04	10	2,5	60	2	1	2	3	14028	12976	2710	2874	2783	3744	8535	6358	5,8	8,7	5,5	12	95	79	1,1	0,7	0,4	0,3	0,5	0,3
P05	7	0	16	3	4	1	3	10227	31759	1769	3737	2928	7153	5529	20868	4,4	6,7	4	6	78	54	0,5	1,3	0,3	0,4	0,3	0,6
P06	7	7	20	1	1	5	5	10533	6906	2136	1457	2533	2471	5863	2979	2,2	6,5	13,6	13	82	80	1,1	0,3	0,3	0,3		
P07	14	7	106	4	4	4	3	14520	11479	3257	3554	3572	3212	7691	4713	3,5	3,5	5,5	7,5	86	80	0,5	0,4	0,3	0,2	0,4	0,2
P08	12	5	40	4	3	4	5	42139	13819	10104	3905	14169	3584	17866	6330	6,9	3,1	7	4	66	87	2,7	0,9	1	0,3	0,7	0,3
P09	9	0,75	20	3	4	2	4	14354	13890	3082	2575	4569	4152	6704	7163	4,6	12,3	11	12	83	49	0,6	0,5	0,4	0,3	0,3	0,2
P10	10	2	19	3	4	4	3	30374	28824	8920	8076	9152	7660	12302	13088	6,5	3,1	8	7,5	60	79	2,1	0,7	1,9	0,6	1,7	0,7
P11	13	0,5	48	5	4	5	4	42062	35150	17316	13202	12420	9528	12326	12419	8,8	6	10	15	85	61	0,8	0,7	0,7	0,6	0,7	0,6
P12	11	0,5	50	4	3	2	3	10532	18428	1897	3546	3038	7137	5597	7746	3,5	4,9	17	14,5	79	61	1,3	1	0,6	0,5	0	0,5

Legend:

- **Total AUC:** Area under the curve for the entire esophagus.
- **Sup AUC:** Area under the curve for the upper third of the esophagus.
- **Mid AUC:** Area under the curve for the middle third of the esophagus.
- **Inf AUC:** Area under the curve for the lower third of the esophagus.
- **ETT:** Esophageal transit time.
- **EET:** Esophageal emptying time.
- **% 10s MAX:** Percentage of radiotracer clearance 10 seconds after peak activity.
- **Ret 10’:** Percentage of radiotracer retention at 10 minutes.
- **Ret 20’:** Percentage of radiotracer retention at 20 minutes.
- **Ret 30’:** Percentage of radiotracer retention at 30 minutes.
